# Top five unanswered questions in plant cell surface research

**DOI:** 10.1016/j.tcsw.2024.100121

**Published:** 2024-02-13

**Authors:** Wout Boerjan, Vincent Burlat, Daniel J. Cosgrove, Christophe Dunand, Paul Dupree, Kalina T. Haas, Gwyneth Ingram, Elisabeth Jamet, Debra Mohnen, Steven Moussu, Alexis Peaucelle, Staffan Persson, Cătălin Voiniciuc, Herman Höfte

**Affiliations:** a Ghent University, Department of Plant Biotechnology and Bioinformatics, Technologiepark 71, 9052 Gent, Belgium; b VIB Center for Plant Systems Biology, Technologiepark 71, 9052 Gent, Belgium; c Laboratoire de Recherche en Sciences Végétales, Université de Toulouse, CNRS, UPS, Toulouse INP, Auzeville-Tolosane, France; d Department of Biology, Pennsylvania State University, University Park, PA 16870, the United States of America; e Department of Biochemistry, University of Cambridge, Cambridge CB2 1QW, UK; f Université Paris-Saclay, INRAE, AgroParisTech, Institut Jean-Pierre Bourgin (IJPB), 78000, Versailles, France; g Laboratoire Reproduction et Développement des Plantes, ENS de Lyon, CNRS, INRAE, UCBL, Lyon, France; h Complex Carbohydrate Research Center and Department of Biochemistry and Molecular Biology, University of Georgia, Athens, GA 30602, USA; i Copenhagen Plant Science Center (CPSC), Department of Plant & Environmental Sciences, University of Copenhagen, Frederiksberg, Denmark; j Horticultural Sciences Department, University of Florida, Gainesville, FL 32611, the United States of America

**Keywords:** Cell expansion, Cell wall architecture, Immunity, Plant cell wall, Polymer structure-function, Polymer synthesis, Signaling

## Abstract

Plant cell wall researchers were asked their view on what the major unanswered questions are in their field. This article summarises the feedback that was received from them in five questions. In this issue you can find equivalent syntheses for researchers working on bacterial, unicellular parasite and fungal systems.

## Introduction

Plant cell wall researchers were asked their view on what the major unanswered questions are in their field. This article summarises the feedback that was received from them in five questions. In this issue you can find equivalent syntheses for researchers working on bacterial, unicellular parasite and fungal systems.

## Question #1: What is the structure–function relationship of cell wall polymers?

*What are the cell wall polymer structures?* Plant cell walls contain a large diversity of polysaccharide, proteoglycan, glycoprotein and phenolic structures. Most polysaccharide types that are common in land plants have been identified but the detailed structures of many polymers remain unknown. This is in part due to the structural heterogeneity of polysaccharides, which challenges current analytical methods, but also to the extraction procedures involving for instance alkaline extraction for cellulose-bound polymers, which removes ester linkages, or the enzymatic release of polysaccharide fragments leading to loss of information on the overall polymer structure. As a result, certain basic questions remain unanswered, such as: (i) how widespread are specific substitution patterns as reported for xylans, xyloglucans or mannans (Yu et al., 2022)? For instance, can the substitutions (methylesterification, acetylation, xylosylation or apiosylation) on homogalacturonans also display specific patterns?; (ii) what is the exact block polymer organisation of cell wall polysaccharides; or: (iii) to what extent are polysaccharides part of proteoglycans as shown for arabinogalactan protein (AGP)-bound pectins? (Mohnen et al., 2024, Tan et al., 2023).

*What is the function of these structures at different scales (molecular, mesoscale, cell wall, cell, organ, plant) and in different abiotic or biotic environments?* Many studies report on correlations between polymer features and functions at different scales. For instance, the presence of RG-I galactan side chains correlates with cell elongation; pectin demethylesterification correlates with the stimulation or inhibition of cell expansion depending on the context (Haas et al., 2021). Causal relations however, are notoriously difficult to unravel even with the help of genetic tools, which otherwise have an excellent track record for the identification of cell wall biosynthetic or modifying enzymes. This is primarily due to the existence of feedback mechanisms, which often obscure causality (question #4). As a result, a clear function for polymer structures could be defined only in a handful of cases. For instance, periodic (methyl)glucuronate substitutions on glucuronoxylans are critical for their interaction with cellulose, by allowing the formation of a 2-fold helix exposing the unsubstituted face to the cellulose microfibril. The same may be true for evenly distributed arabinofuranosyl residues on glucuronoarabinoxylans (GAX) in grasses or substitution patterns of xyloglucans and glucomannans. Interestingly, the substitution pattern, rather than the exact chemical nature of the substituent appears to be the more critical parameter for the interaction with cellulose (Xiong et al., 2015). Similarly, despite their different chemical nature, mannans and xyloglucans appear to be partially inter-exchangeable *in vivo* (Grieß-Osowski and Voiniciuc, 2023). This raises questions about the extent to which the chemical make-up of hemicellulose backbones (mannans, xyloglucans or xylans) and their decorations affect binding to cellulose and cell wall properties. Another example is RG-II, the complex primary structure of which appears to support the formation of a disc-like 3D structure that supports dimerization through the formation of boron diester bonds. Boron deprivation revealed that this dimerization provides crosslinks that are essential to maintain the integrity of the cell wall (Pérez et al., 2003). Another important unanswered question concerns the function of the diverse hydroxyproline rich glycoprotein (HRGP) polymers. The recent findings that most RG-I in the wall appears to be covalently attached to AGPs (Tan et al., 2023) and that Leucine Rich Repeat eXtensin-Rapid ALkalinization Factor (LRX-RALF) complexes structure the cell wall through binding to pectins (Moussu et al., 2023, Moussu and Ingram, 2023), suggest that other HRGPs, like extensins or proline-rich proteins may play equally important roles in the cell wall (Silva et al., 2020; Moussu and Ingram, 2023, Moussu et al., 2023).

Likewise, it is largely unknown to what extent lignin composition affects the structural properties of the cell wall. Lignin is a polymer derived from radical coupling of *p*-coumaryl (generating the H unit in the polymer), coniferyl (generating the G unit) and sinapyl alcohol (generating the S unit), whose composition varies among taxa, cell types and upon environmental stimuli (Cesarino, 2019; Vanholme et al., 2019). However, how the 3-D structure of the lignin polymer is affected by compositional differences, and how the structural variation brings about new properties to the cell wall is largely unknown, although progress in this area shows that specific lignin chemistries in different types of tracheary elements entail differences in cell wall biomechanics and hydraulic properties (Ménard et al., 2022). It is also increasingly recognized that lignin is composed of more units than those engendered by coupling of *p*-coumaryl, coniferyl and sinapyl alcohol. In addition to monolignol biosynthesis pathway intermediates, such as cinnamaldehydes, various monolignol esters are also part of the polymer, e.g. sinapyl-*p*-hydroxybenzoate in poplar or sinapyl *p*-coumarate in grasses. Even flavonoids, hydroxystilbenes and tyramine ferulates have been detected as part of the lignin polymer, depending on the species (del Río et al., 2022). In seed coats of certain species such as the vanilla orchid, lignin is made from caffeoyl alcohol (C-lignin). It is unknown what biological roles these additional units have and how they affect lignin properties. In this respect, it is important to consider that monolignols, dilignols and a range of other molecules can diffuse through the plasma membrane without the need for transporters, implying that even molecules that have no biological role to play in the cell wall, but which can diffuse through a lipid bilayer and be oxidized by laccase or peroxidases, can be coupled into the lignin polymer (del Río et al., 2022). The complex nature of the lignin polymer, which is generated by combinatorial radical chemistry and which therefore lacks a predefined 3-D structure, makes it difficult to relate structural differences to cell wall properties. Such new properties can be studied in mutants or natural tissues with extreme H/G/S/C lignin compositions.

The function of most other polymer structures also remains unknown. Determining *in vivo* function will require the elucidation of the biosynthetic steps (question #5); methods for their manipulation *in vivo* and the observation of the induced changes in cell wall and macroscopic properties at short time scales to avoid compensatory changes (question #4) and a better understanding of cell wall architectures in different cell types combined with multiscale computational modelling to reveal emergent properties of polymer assemblies (question #2).

## Question #2: What is the wall architecture and how is this assembled in distinct species and cell types at different growth stages in changing environments?

*What is the cell wall architecture?* The challenge here is to build models for the architecture of different cell wall types, which combine information on the spatial distribution of polymer structures and how they are connected to each other to predict physicochemical properties of the cell walls.

New insights in molecular interactions among polymers have been obtained in particular by using 2D solid state NMR on never-dried walls, *in vitro* interaction studies and molecular dynamics (MD) modelling of the interactions between cellulose and xyloglucan/xylan for instance. Information on the mesoscale organisation of cellulose microfibrils and their movements during wall extension was obtained from atomic force microscopy (AFM) studies that revealed extensive cellulose bundling and ramifying networks (Zhang et al., 2017). Based on such observations in onion external epidermal walls, a coarse-grained MD model allowed complex mechanical properties of the cell wall to be predicted from lateral contacts between cellulose microfibrils, which form a coherent network that carried most of the wall stress (Zhang et al., 2021). Cryo-electron tomography on the same cell walls showed a reticulated pectin matrix and microfibrils in successive layers with a bimodal angular pattern ±45° relative to the cell’s long axis (Nicolas et al., 2022). Super-resolution dSTORM microscopy with anti-glycan antibodies showed in anticlinal leaf epidermal cell walls that homogalacturonans existed both as a part of an unordered network and as filaments oriented perpendicularly to the cell surface (Haas et al., 2020). A network of demethylesterified homogalacturonan filaments formed by a pectin-binding LRX8-RALF4 complex was observed in pollen tube cell walls (Moussu et al., 2023, Moussu and Ingram, 2023). This complex exerts a condensing effect, thus patterning the cell wall polymers into a reticulated network critical for cell wall integrity and expansion during pollen tube growth.

Important open questions are: What is the exact crystal arrangement of the glucan chains in cellulose microfibrils? Are there always 18 glucan chains arranged into sheets with respectively 2,3,4,4,3 and 2 chains per sheet, or is this variable between cells or species and during the life of the cell? This is important information for the modelling of the cellulose-hemicellulose interactions. What are the covalent and non-covalent crosslinks among polysaccharides, proteins and phenolics and what are their roles in cell wall architecture and mechanics?

*How are walls assembled?* The assembly of the cell wall polymer network combines the physical properties of the distinctive polymers in the wall (Cosgrove, 2022) with cellular processes (synthesis and secretion/endocytosis of polymers and wall modifying agents, control of surface pH, calcium levels, redox state), which regulate physicochemical properties and assembly kinetics of the polymers in the apoplastic space. Interestingly, transient polymer structures are observed during the assembly process, e.g. pectin turnover appears to be important during cell wall assembly (Nicolas et al., 2022) and unbranched RG-I chains that accumulate in the seed mucilage of Arabidopsis appear to be derived from highly branched precursors (Williams et al., 2020).

Cellulose orientation in the cell wall depends to a large extent on the dynamics of microtubules, which control delivery to and retrieval from the cell surface of cellulose synthase complexes and guide their trajectories in the plasma membrane. Several components of the machinery controlling the cellulose synthase complex (CSC) interaction with cortical microtubules have been identified (McFarlane et al., 2014). Microtubule orientation is part of a feedback loop orienting microtubules along the principal stress vector in the tissue, which depends at least in part on the orientation of the cellulose microfibrils. How microtubules sense the stress direction is not known (Coen and Cosgrove, 2023, Hamant et al., 2019).

*Other unanswered questions are*: What is the cellular mechanism underlying the spatial periodicity observed in many cell walls, ex. distinctive angular microfibril patterns in successive layers of certain cell walls? What controls the polymer stoichiometry in the cell wall, e.g. are there mechanisms that coordinate cellulose synthesis at the plasma membrane with the synthesis, secretion and/or turnover of matrix polymers? What is the role of transient polysaccharide structures and protein-polysaccharide interactions in cell wall assembly and expansion? Can we distinguish cell wall micro-or nanodomains in different cell wall types (Dauphin et al., 2022)? Are there specific spatial cues that determine the deposition of cell wall components, or do domains arise spontaneously for instance through phase separation?

## Question #3: How do the physical properties of the wall relate to wall expansion?

The widely accepted view is that cell expansion is turgor-driven and limited by cell wall relaxation, mediated for example by the wall-loosening action of expansins (Cosgrove, 2022). Mechanical connectivity between cells with different wall properties can give rise to internal growth conflicts and tissue tensions that may play an integral role in plant morphogenesis (Coen and Cosgrove, 2023). In this context, it is commonplace today to use elastic modulus as a stand-in for wall extensibility, in the growth sense, yet the theoretical basis for this assumption has been questioned and dynamic measurements of growth rate show that it can stop within seconds, while changes in wall elasticity and viscoelasticity are much slower, if they occur at all. How do we reconcile such observations? A possible answer to this paradox may come from a better appreciation of the role of wall material deposition in the growth process. A critical role for cell wall secretion/insertion in growth rate control has been proposed for tip-growing root hair cells and pollen tubes (as for fungal hyphae) and for cells of the Charophyte *Chara corallina* (Peaucelle et al., 2012). A turgor-independent motor for cell expansion, in which oriented pectin filaments radially expand upon demethylesterification, was proposed to explain lobe formation in puzzle-shaped epidermis cells (Haas et al., 2020). How wall synthesis, expansion and turgor-driven relaxation interact in the control of cell expansion remains a major unanswered question.

Another critical related parameter is cell wall hydration. How does the water content evolve during cell wall expansion, differentiation and in environments with changing water availability and how is this controlled? How does this affect polymer interaction strength, cell wall porosity and viscoelasticity, the activity of cell wall modifying proteins and growth? How do the substitution patterns and enzymes in turn affect the hydration of the cell wall? In this context it will also be interesting to compare the wall architecture in plant species growing in fresh water, on land or in salt water.

Tropic movements (stem bending or straightening) in woody stems are driven by secondary growth involving wood and/or bark. Wood fibers in reaction wood in many angiosperm species have a so-called gelatinous (G) layer, consisting of highly crystalline cellulose oriented along the fiber axis in a RG-I-rich matrix (Sivan et al., 2024). The interaction between RG-I and cellulose appears to play an active role in stem bending and straightening (Gorshkova et al., 2018). How exactly this creates the massive forces required for these events is still largely an unanswered question.

Answering these questions will require more interdisciplinary research combining biology, biophysics and computational modeling.

## Question #4: How does the cell wall contribute to signalling?

Increasing evidence supports a critical role for cell walls in signalling during growth, development, immunity and abiotic stress responses (Wolf, 2022). Little is known however on the respective contribution of chemical and physical cues, the receptors and mechanosensors involved, the signalling networks, the critical responses that lead to cell wall changes and the integration of these processes in growth, development and adaptation to the environment.

Cell walls can be a source of chemical cues such as the accumulation of altered polymer epitopes, polymer precursors or degradation products (e.g. oligosaccharides) recognized by specific receptors. Cell walls can also contribute to physical cues associated with stress-induced nanoscale strains. The latter may involve mechanosensitive channels or strain-induced changes in conformation, and/or ligand binding affinity, of cell surface proteins/polysaccharides. Other physical cues such as changes in cell wall hydration, pH or ion balance may also play a role (Wolf, 2022).

An interesting emerging theme is the impact of peptide signalling on the cell wall and vice versa. The genomes of land plants encode hundreds to thousands of potential secreted signalling peptides, but their function and perception mechanisms are still mostly unknown, especially outside of model plant species such as *A. thaliana*. “Can we identify these peptides *in situ* and their functions and corresponding receptors?” is a mostly unanswered question. Cell wall cues may also indirectly affect signalling through promoting membrane receptor complex association in plasma membrane nanodomains (Dauphin et al., 2022). In addition, cell wall composition/structure may affect the diffusion of signalling peptides and inversely, apoplastic (chemical or mechanical) cues may feed back on signalling via cell wall modifications (Dauphin et al., 2022). Finally, the interaction of signalling peptides with cell wall polymers may also directly affect the physico-chemical properties of the cell wall (Moussu et al., 2023, Moussu and Ingram, 2023).

Concerning biotic interactions, the cell wall is the theatre of an evolutionary enzymatic arms race among plant hosts and microorganisms, in which pathogens develop increasingly sophisticated strategies to penetrate and disassemble the host’s cell walls, while avoiding being noticed by the immune system, and hosts develop ever more complex strategies to generate and respond to elicitor molecules derived from the pathogen itself or their own walls in response to the microbe (Wolf, 2022). Conversely, the plant cell wall can undergo subtle modifications to accommodate symbiotic associations (Su, 2023). Unanswered questions in this context are what are the processes involved and how can this knowledge lead to new crop protection and symbiosis-promoting strategies.

## Question #5: How are cell wall synthesis and modification regulated?

*What are the enzymes?* Genetic analysis and enzymology on heterologously expressed proteins has identified enzyme families for the synthesis of most polysaccharide backbones. The enzymes for many side chain additions however, are still missing (Amos and Mohnen, 2019). Likewise, lignin biosynthesis enzymes are often encoded by gene families. Some family members appear to be involved in developmental lignin deposition whereas others in the deposition of stress lignin (Cesarino, 2019). The fluxes through the different branches and parallel routes (e.g. towards caffeic acid) in the monolignol biosynthesis pathway differ between species, cell types and environmental conditions. It is largely unknown however, whether these different fluxes are needed to divert pathway intermediates into different classes of secondary metabolites, or whether they are needed to adapt lignin structure to serve particular structural needs of the walls of different cell types, or both.

*How are the cell wall biosynthetic enzymes distributed within Golgi bodies*? With the exception of cellulose, callose and perhaps mixed-linked glucans, polysaccharide biosynthesis mostly takes place within Golgi bodies. How are enzymes partitioned in cis-, medial-, and trans-Golgi, how are they transferred and inserted into the membrane and how is membrane trafficking regulated are largely unanswered questions. In addition, it remains to be determined how substrate, metal and ion concentrations, and pH in the different compartments affect specific polymer synthesis (Hoffmann et al., 2021).

*What are the roles of “cell wall metabolons”?* A metabolon is a complex of sequential enzymes in a metabolic pathway. Protein complexes that may work as components of metabolons were identified for cellulose (McFarlane et al., 2014), xyloglucan and pectin (Amos and Mohnen, 2019) and monolignol synthesis (Gou et al., 2018). For most other polymer biosynthetic enzymes, we don’t know how they engage with other proteins. The identification of cell wall metabolons will be instrumental for the synthesis of tailored polysaccharides *in vitro* or in heterologous systems.

*How are cell wall synthesis/modification transcriptomes and proteomes regulated?* We know quite a deal about the control of secondary wall synthesis/modifications, but virtually nothing for primary cell walls. Most of the transcription factors found to directly regulate the expression of cell wall-related enzymes affect the synthesis of multiple cell wall components (e.g. MYB46), so additional genetic strategies to specifically adjust only one type of polymer are still needed.

*How are polymer modifications in the cell wall regulated?* Plant cell walls contain hundreds of cell wall modifying proteins (San Clemente et al., 2022). How they are regulated in the intercellular space is poorly understood. This may involve their secretion and proteolytic maturation, regulation by pH, redox potential, calcium, but also through their compartmentalization in cell wall subdomains as a result of targeted secretion and/or interactions with cell wall polymers (Dauphin et al., 2022). All these activities are most likely fine-tuned through feedback processes, which remain to be discovered.

*How do oxidoreductases regulate both cell wall stiffening and loosening?* Among the cell wall enzymes exist a large number of oxidoreductases with opposite effects on cell wall dynamics due to their stiffening (through polymer crosslinking) or loosening (through polymer scission) actions (Francoz et al., 2015). Molecular mechanisms controlling the anchoring of individual peroxidases to cell wall subdomains are emerging (Dauphin et al., 2022) but how the opposite activities are balanced remains poorly understood.

*What is the involvement of dirigent proteins in lignan and lignin biosynthesis?* Recent data show that dirigent proteins family members are able to oxidize coniferyl alcohol to the three dilignols (8-O-4′, 8-5′ and 8-8′ coupled) in the presence of hydrogen peroxide. They play an essential role in the localized deposition of lignin in the Casparian strip in the root endodermis (Gao et al., 2023). Whether dirigent proteins play a role in lignin deposition in general is an important next question to be answered. It is also largely unclear what the role of lignans (stereo specifically coupled monolignol dimers and their derivatives) is in lignification and their mode of action.

*What is the nature of the initiation sites of lignin polymerization*? Is the initiation of lignin polymerization controlled by localized monolignol oxidation? And do certain cell types need specific molecules to initiate lignin polymerization?

## Conclusions

Many of these unanswered questions may find answers in the coming years thanks to recent, often spectacular, technological advances. These include the synthesis of defined polymers *in vitro* or in heterologous hosts (Pauly et al., 2019), new techniques for the direct observation of individual polysaccharides and proteoglycans using low-temperature scanning tunnelling microscopy (Anggara et al., 2023), nanopore sequencing (Cai et al., 2021), improved mass spectroscopy for post-translational modification analysis (Ytterberg and Jensen, 2010), super-resolution microscopy combined with nanobodies (Haas et al., 2020) and metabolic click labelling (Ropitaux et al., 2022), and new biomimetic systems to study interactions among polymers *in vitro* (Villares et al., 2015). Advances that will certainly impact our understanding of enzymatic synthesis and modification of cell wall polymers include methods that allow modeling of the biosynthetic and remodelling enzymes, such as Alphafold and dynamic simulations and molecular modeling, and enable prediction of protein:protein and protein:glycan interactions and quaternary structure. This together with evermore sensitive techniques for the dynamic manipulation and observation of wall rheology (Elsayad et al., 2016, Michels et al., 2020) and advances in multiscale computational modelling (Zhang et al., 2021) will provide valuable information on polymer structure–function relationship, cell wall architecture, assembly and expansion, signaling and regulation. These advances may pave the way for the design of new bioinspired nanomaterials and new strategies for environmentally-friendly crop protection and the breeding of crops better adapted to industrial processes, biofuel production and a changing climate.

## CRediT authorship contribution statement

**Wout Boerjan:** Writing – review & editing, Writing – original draft. **Vincent Burlat:** Writing – review & editing. **Daniel J. Cosgrove:** Writing – review & editing. **Christophe Dunand:** Writing – review & editing. **Paul Dupree:** Writing – review & editing. **Kalina T. Haas:** Writing – review & editing. **Gwyneth Ingram:** Writing – review & editing. **Elisabeth Jamet:** Writing – review & editing. **Debra Mohnen:** Writing – review & editing. **Steven Moussu:** Writing – review & editing. **Alexis Peaucelle:** Writing – review & editing. **Staffan Persson:** Writing – review & editing. **Cătălin Voiniciuc:** Writing – review & editing. **Herman Höfte:** Writing – original draft, Conceptualization.

## Declaration of competing interest

The authors declare that they have no known competing financial interests or personal relationships that could have appeared to influence the work reported in this paper.

## Data Availability

No data was used for the research described in the article.
